# Supplementary figures and images for: Emergence of Assortative Mixing between Clusters of Cultured Neurons
Source: PLoS Comput Biol. 2014 Sep 4;10(9):e1003796. doi: 10.1371/journal.pcbi.1003796 (PMC4154651; doi:10.1371/journal.pcbi.1003796)

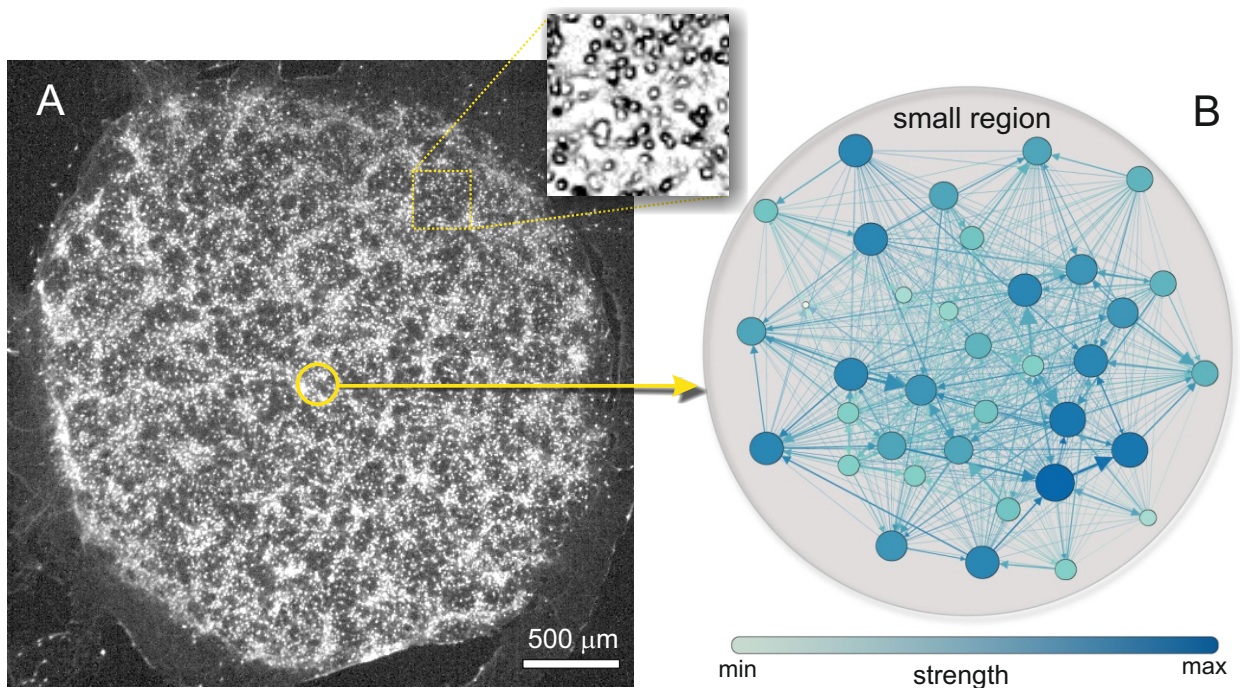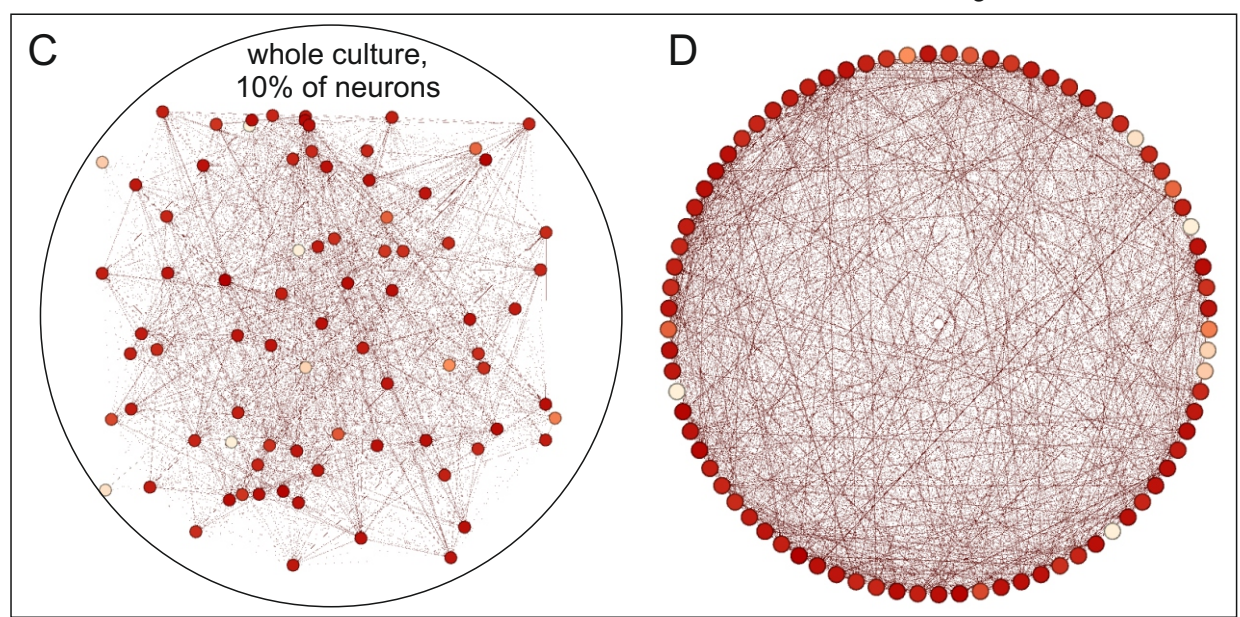

Supplement: Figure S1 — Homogeneous cultures. A The seeding of neurons in cover glasses previously coated with poly-l-lysine gives rise to neuronal cultures with a quasi-homogeneous distribution of neurons. A typical circular culture in diameter contains about neurons that can be well identified either as bright spots in the fluorescence recordings or as circular objects in bright-field images (small panel on the right). Spontaneous activity in these homogeneous cultures is typically recorded at frames/s, which suffices to extract the time delays between consecutive neuronal activations. The particular experiment shown here corresponds to network ‘P’ of the main text, with a total of neurons manually selected over the images and monitored along . The analysis of their spontaneous activity traces is analyzed in the context of our model, finally procuring the functional connectivity network and its topological properties. B Given the large number of nodes analyzed and the high average degree of the resulting network ( functional connections per neuron), a representation of the complete functional network is unpractical. As an example of the obtained functional networks, we here show all the functional links within a small region placed in the center containing neurons. Connections are both color and thickness coded according to their weight. Nodes are color coded according to their strength. C As an alternative representation, we show here a of the population ( neurons randomly chosen), each neuron showing the of its links. Nodes and links are color coded according to their strength and weight, respectively. D A ring graph of the same neurons shows that most of them display a similar connectivity, in contrast to the strong modularity and variability in connectivity exhibited by the clustered cultures. (PDF) [file pcbi.1003796.s001.pdf]

# Dependence of the variance $C$ on culture properties

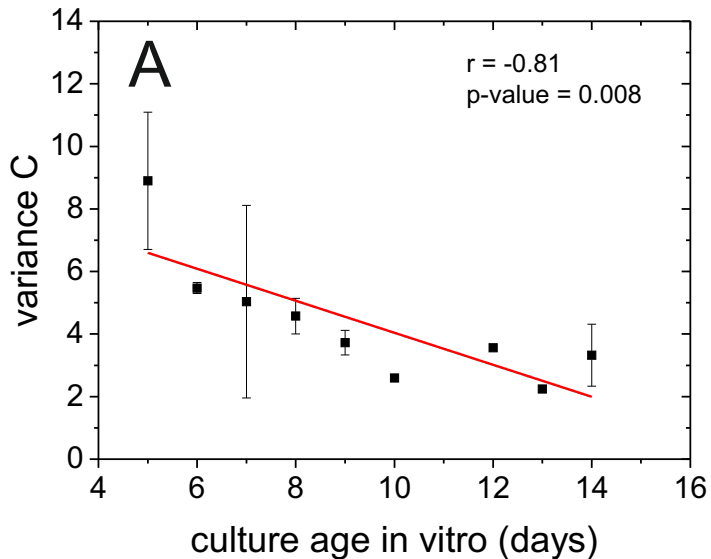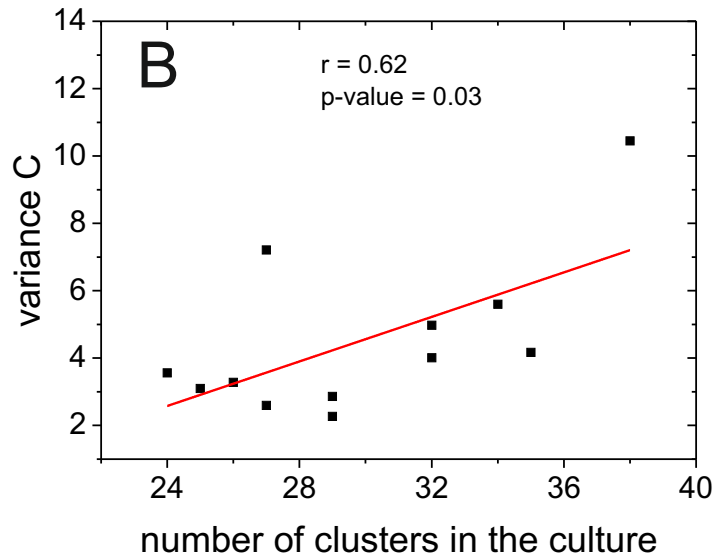

Supplement: Figure S2 — Variance and culture properties. A The variance is obtained from the Gaussian fit of the activation delays between pairs of clusters. The plot shows that decreases with the culture age in vitro, indicating that young cultures display a slower dynamics (larger delay times and therefore larger variance) than mature cultures. B The variance increases with the number of clusters in the culture, indicating a broader and richer distribution of time delays as more clusters participate in the dynamics of the network. Errors bars in A show standard deviation. (PDF) [file pcbi.1003796.s002.pdf]

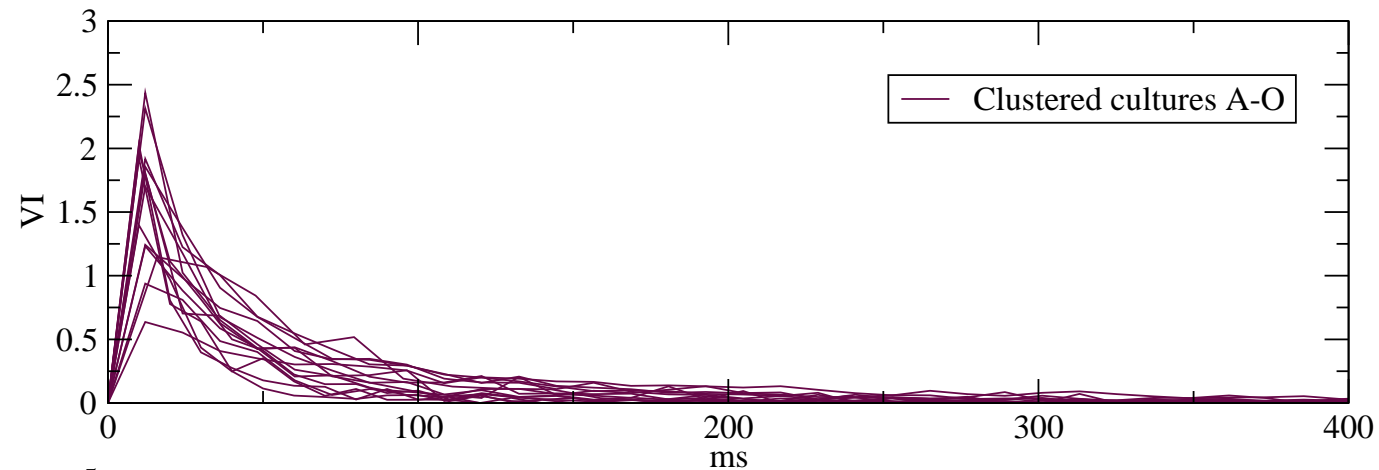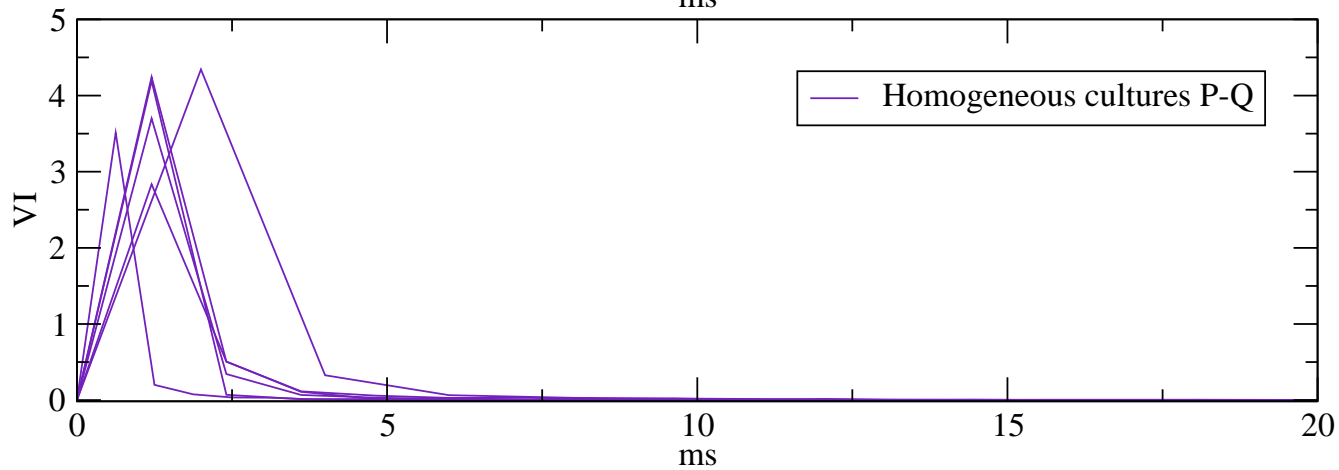

Supplement: Figure S3 — Sensitivity of the functional network construction to the cut-off times. The cut-off determines the end of a sequence and therefore its variation modifies the set of burst chosen. The cut-off is set to for clustered cultures and for homogeneous ones. To assess the sensitivity of the grouping of bursts to the cut-off, we have computed the Variation of Information (VI) between the grouping of bursts at a certain cut-off value and the previous one. It is computed as , where and are two partitions, is the entropy and is the mutual information. In our case, each partition is the set of burst found at a certain value of the cut-off. We have screened the cut-off values from to . The analysis shows that for homogeneous and for clustered cultures are values for which the variation of information is already stabilized. Thus, the modification of the cut-off within the stabilization region does not change the grouping of clusters in each burst, and therefore the derived functional networks, as well as the corresponding network measures, remain the same. (PDF) [file pcbi.1003796.s003.pdf]

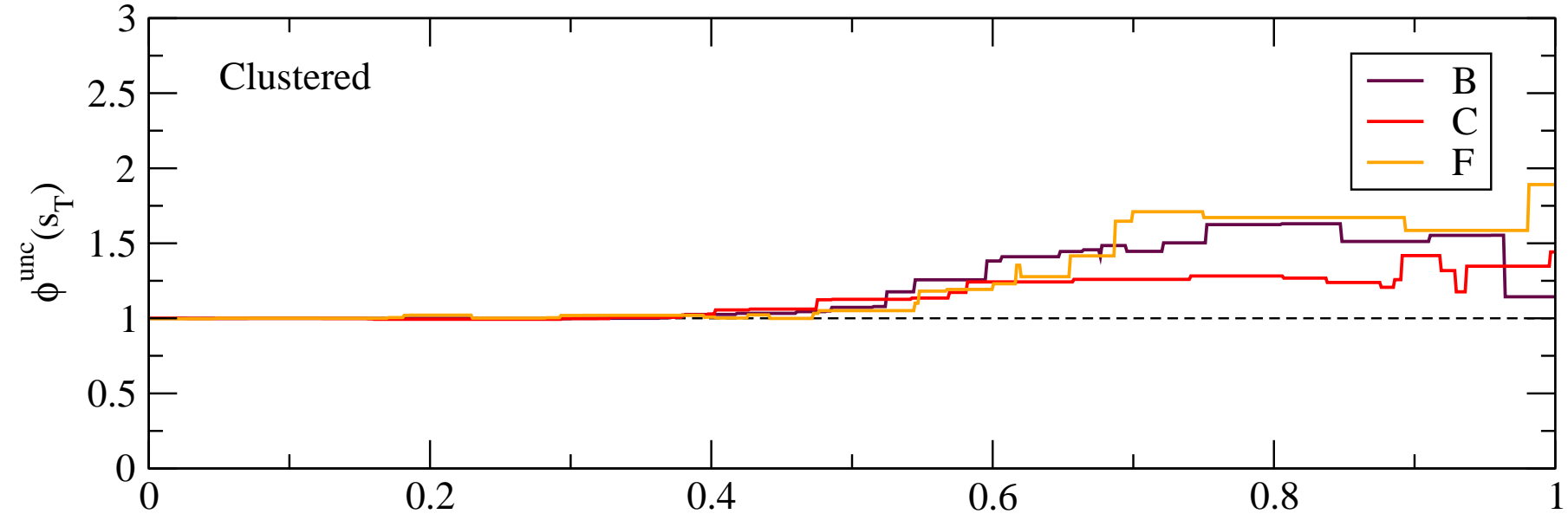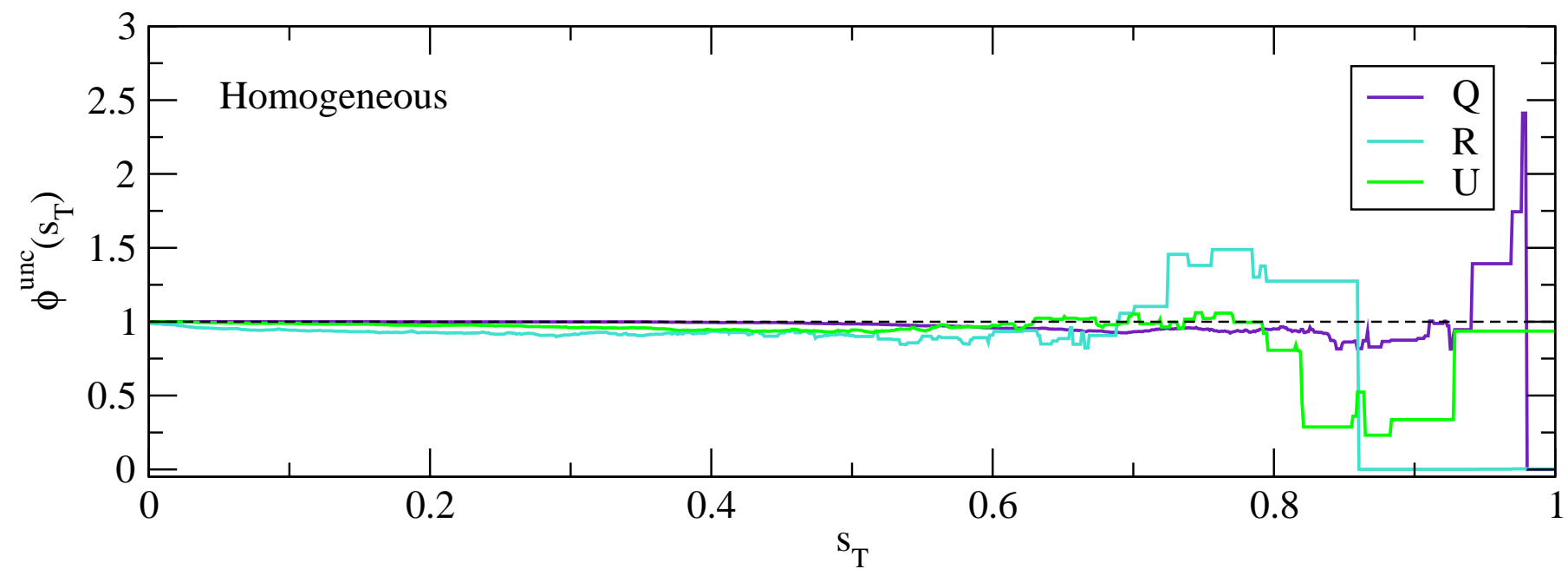

Supplement: Figure S5 — ‘Rich-club’ analysis. The evaluation of the rich-club is performed by computing the ratio between the connectivity strength of highly connected nodes and its randomized counterpart, , and for gradually larger values of the strength threshold . The figure shows the rich-club analysis for 3 representative clustered and homogeneous cultures. Clustered networks exhibit values of systematically higher than for large values of the strength threshold , evidencing the existence of a rich-club core of highly connected clusters in the network. On the contrary, homogeneous cultures display a mixture of positive and negative values, and with an average around , ruling out the existence of the rich-club property. (PDF) [file pcbi.1003796.s005.pdf]
