# Supplementary material for: Emergence of Assortative Mixing between Clusters of Cultured Neurons
Source: PLoS Comput Biol. 2014 Sep 4;10(9):e1003796. doi: 10.1371/journal.pcbi.1003796 (PMC4154651; doi:10.1371/journal.pcbi.1003796)
Supplement: Figure S4 — Relation between the structural traits of the network and the functional connectivity. A Dependence of the weight of the functional connections on the width of physical connections between directly connected clusters. The sketch conceptually shows the comparison between structural and functional links. The plot represents the analysis of pairs of clusters, with data binned for similar widths. No significant correlation is observed. B The dependence of the node strength on cluster size shows no correlation, indicating that the functional connectivity cannot be assessed from the size of the clusters. Data is based on the analysis of clusters. C For the same clusters, this plot shows that the activity of a cluster is independent of its size. D Activity within a burst is always initiated by a particular cluster, which triggers the sequential activation of all the downstream clusters. To quantify the importance of these ‘initiators of activity’ in network dynamics we computed the number of times that a cluster of a given size initiates a sequence of activations. The plot shows that there is no a significant correlation between initiation and size. The analysis is based on the study of bursts. All these results indicate that the functional connectivity cannot be drawn from a visual inspection of the neuronal culture. Errors bars show standard deviation. (PDF) [file pcbi.1003796.s004.pdf]

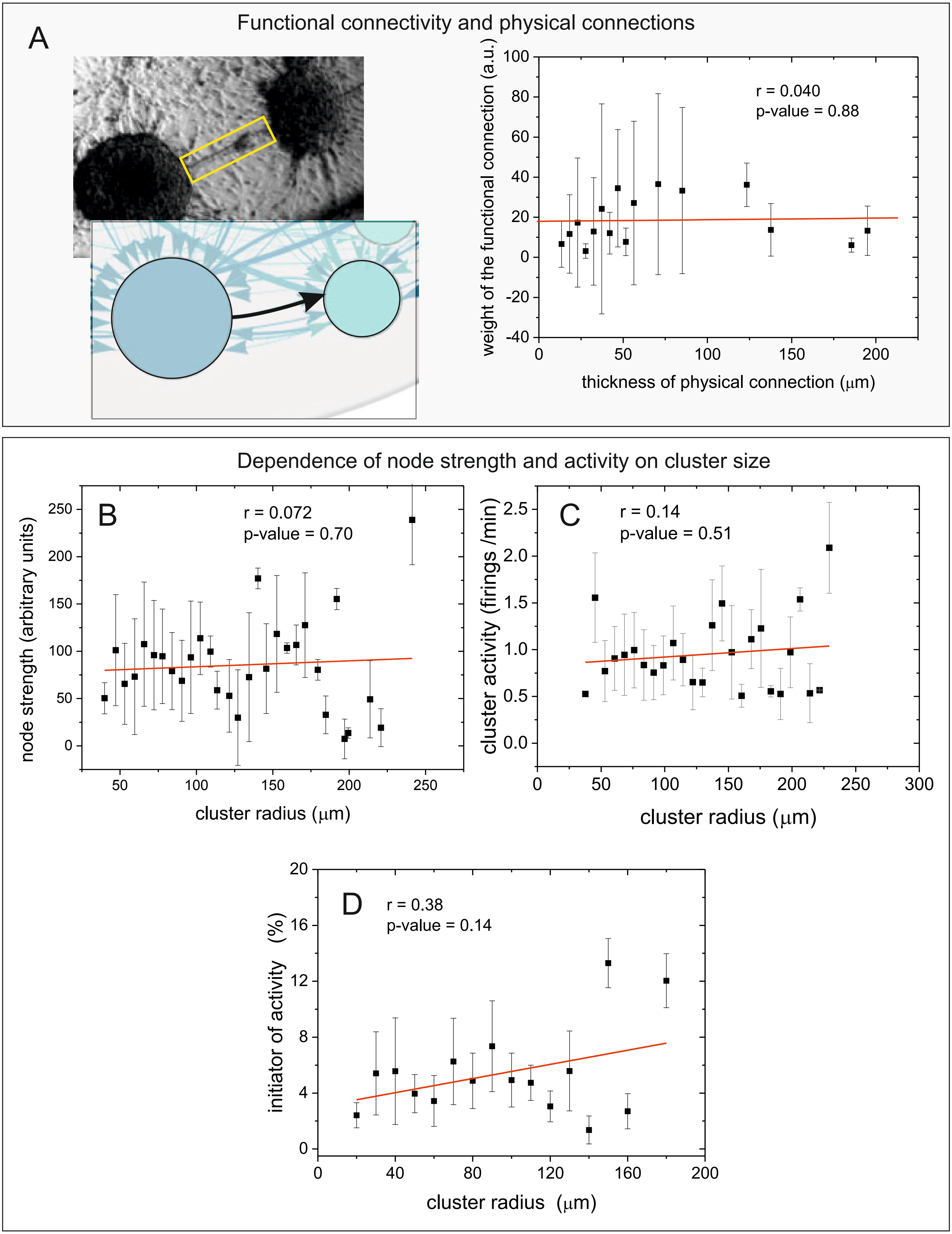

Functional connectivity and physical connections
A
r = 0.040
p-value = 0.88
weight of the functional connection (a.u.)
thickness of physical connection (μm)
100
80
60
40
20
0
-20
-40
0
50
100
150
200
Dependence of node strength and activity on cluster size
B
r = 0.072
p-value = 0.70
node strength (arbitrary units)
cluster radius (μm)
250
200
150
100
50
0
C
r = 0.14
p-value = 0.51
cluster activity (firings /min)
cluster radius (μm)
2.5
2.0
1.5
1.0
0.5
0.0
300
D
r = 0.38
p-value = 0.14
initiator of activity (%)
cluster radius (μm)
20
16
12
8
4
40
80
120
160
