# Supplementary material for: Emergence of Assortative Mixing between Clusters of Cultured Neurons
Source: PLoS Comput Biol. 2014 Sep 4;10(9):e1003796. doi: 10.1371/journal.pcbi.1003796 (PMC4154651; doi:10.1371/journal.pcbi.1003796)
Supplement: Figure S6 — Control experiment. A Bright field image of a clustered network whose spontaneous activity has been recorded for . The average bursting rate of the network is . B Corresponding functional network. The size of the nodes is proportional to the size of the actual clusters, and their color is proportional to their strength. The weights of the links are both color and thickness coded. The darker the color, the higher the value of the observable. C Analysis of the 2 h recording in three blocks, in duration each, and containing bursts. The blocks show very similar traits between them, as well as with the entire recording. The blocks exhibit similar assortativity values, and share both the most important links and nodes’ strengths. indicates the assortativity value of the depicted network, averaged over the Pearson and Spearman formulations. (PDF) [file pcbi.1003796.s006.pdf]

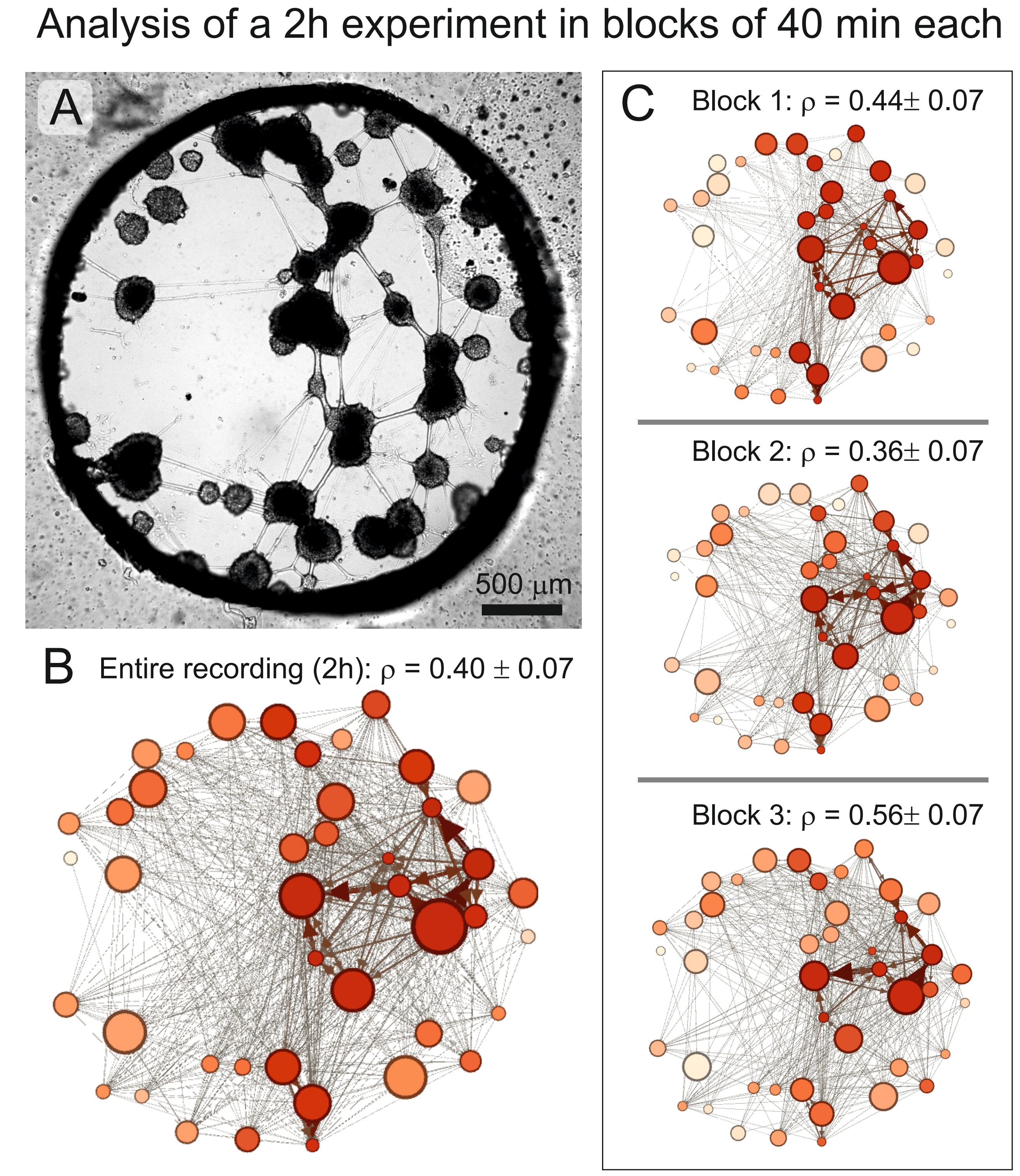

Analysis of a 2h experiment in blocks of 40 min each
A
500 μm
B
Entire recording (2h): ρ = 0.40 ± 0.07
C
Block 1: ρ = 0.44± 0.07
Block 2: ρ = 0.36± 0.07
Block 3: ρ = 0.56± 0.07
